# Supplementary material for: The SGLT2 inhibitor empagliflozin improves cardiac energy status via mitochondrial ATP production in diabetic mice
Source: Commun Biol. 2023 Mar 17;6:278. doi: 10.1038/s42003-023-04663-y (PMC10023657; doi:10.1038/s42003-023-04663-y)
Supplement: Supplementary file 2 — Supplementary Figures [file 42003_2023_4663_MOESM2_ESM.pdf]

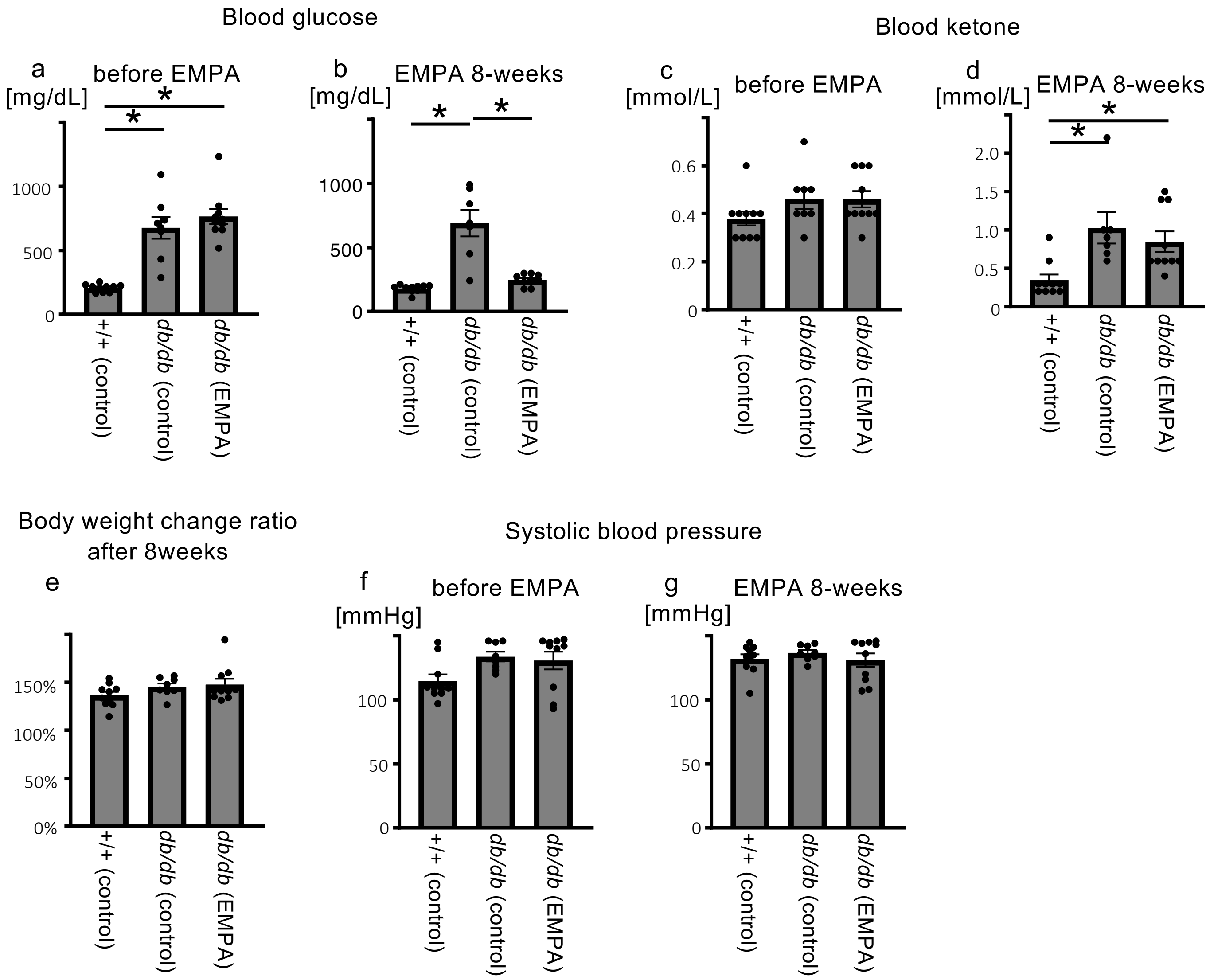

Supplementary Figure 1. Effects of chronic treatment with empagliflozin on systolic blood pressure, blood ketone, blood glucose and body weight in cytoATP-Tg mice. a-b Graph of the blood glucose before treatment with empagliflozin (**a**) (+/+ [control]: *n* = 10, *db*/*db* [control]: *n* = 8, *db*/*db* [EMPA]: *n* = 10; EMPA: empagliflozin) and after 8-week treatment with empagliflozin (**b**) (+/+ [control]: *n* = 10, *db*/*db* [control]: *n* = 8, *db*/*db* [EMPA]: *n* = 10). *p = 0.000. **c-d** Graph of the blood ketone before treatment with empagliflozin (**c**) (+/+ [control]: *n* = 10, *db*/*db* [control]: *n* = 8, *db*/*db* [EMPA]: *n* = 10) and after 8-week treatment with empagliflozin (**d**) (+/+ [control]: *n* = 10, *db*/*db* [control]: *n* = 8, *db*/*db* [EMPA]: *n* = 10). *p = 0.005, 0.004 each. **e** Graph of the body weight change ratio after 8 weeks (+/+ [control]: *n* = 10, *db*/*db* [control]: *n* = 8, *db*/*db* [EMPA]: *n* = 10). **f-g** Graph of the systolic blood pressure before treatment with empagliflozin (**f**) (+/+ [control]: *n* = 10, *db*/*db* [control]: *n* = 8, *db*/*db* [EMPA]: *n* = 10) and after 8-week treatment with empagliflozin (**g**) (+/+ [control]: *n* = 10, *db*/*db* [control]: *n* = 8, *db*/*db* [EMPA]: *n* = 10).

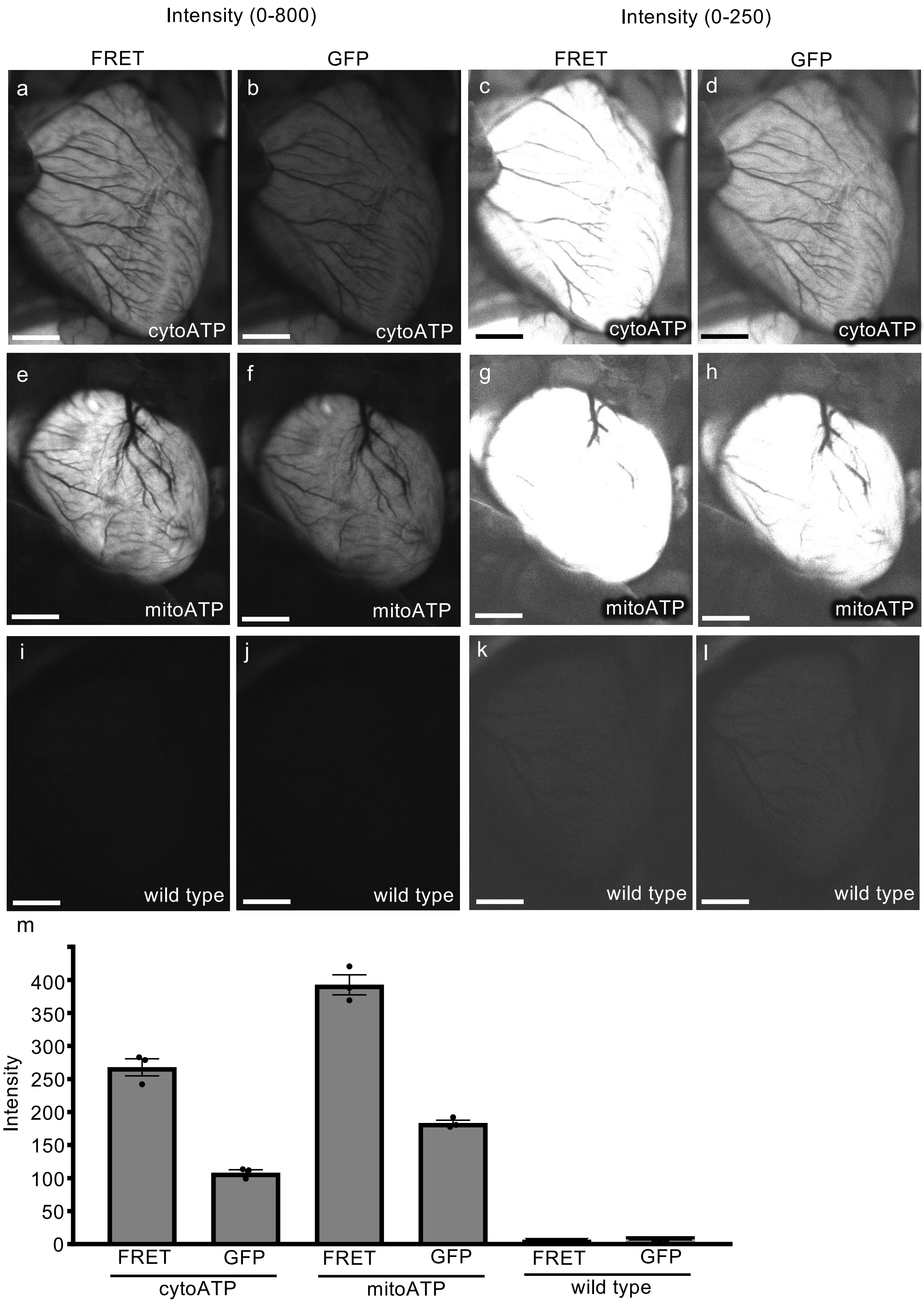

Intensity (0-800)
Intensity (0-250)
FRET
GFP
FRET
GFP
a
cytoATP
b
cytoATP
c
cytoATP
d
cytoATP
e
mitoATP
f
mitoATP
g
mitoATP
h
mitoATP
i
wild type
j
wild type
k
wild type
l
wild type
m
Intensity
0
50
100
150
200
250
300
350
400
FRET
GFP
FRET
GFP
FRET
GFP
cytoATP
mitoATP
wild type

Supplementary Figure 2. Autofluorescence in the hearts did not have a significant effect on measurement of FRET and GFP. **a-d** Fluorescence intensity of FRET (**a, c**) and GFP (**b, d**) in a live heart of a cytoATP-Tg mouse (FRET, Fluorescence Resonance Energy Transfer). Higher brightness indicates stronger intensity. Scale bar: 2 mm. **e-h** Fluorescence intensity of FRET (**e, g**) and GFP (**f, h**) in a live heart of a mitoATP-Tg mouse. Higher brightness indicates stronger intensity. Scale bar: 2 mm. **i-l** Autofluorescence intensity of FRET (**i, k**) and GFP (**j, l**) in a live heart of a wild-type mouse. Higher brightness indicates stronger intensity. Scale bar: 2 mm. **m** Graph of the intensity of FRET and GFP in each mouse (cytoATP: n = 3, mitoATP: n = 3, wild type: n = 3).

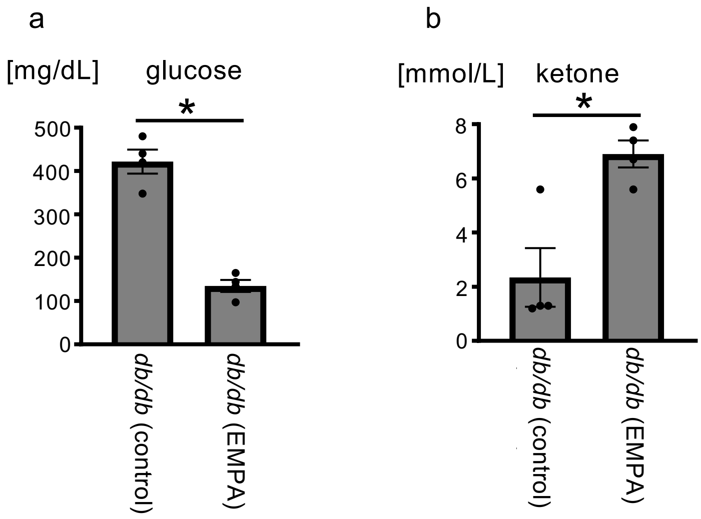

Supplementary Figure 3. Effects of single treatment with empagliflozin on blood glucose and blood ketone in *db/db*; mitoATP-Tg mice. a-b Graph of the blood glucose (**a**) and ketone (**b**) levels in *db/db*; mitoATP-Tg mice after 4 h of fasting followed by 3 h of control or empagliflozin (30 mg/kg b.w., EMPA) treatment (*db*/*db* [control]: $n = 4$, *db*/*db* [EMPA]: $n = 4$; EMPA: empagliflozin). *p = 0.000, 0.009 each.

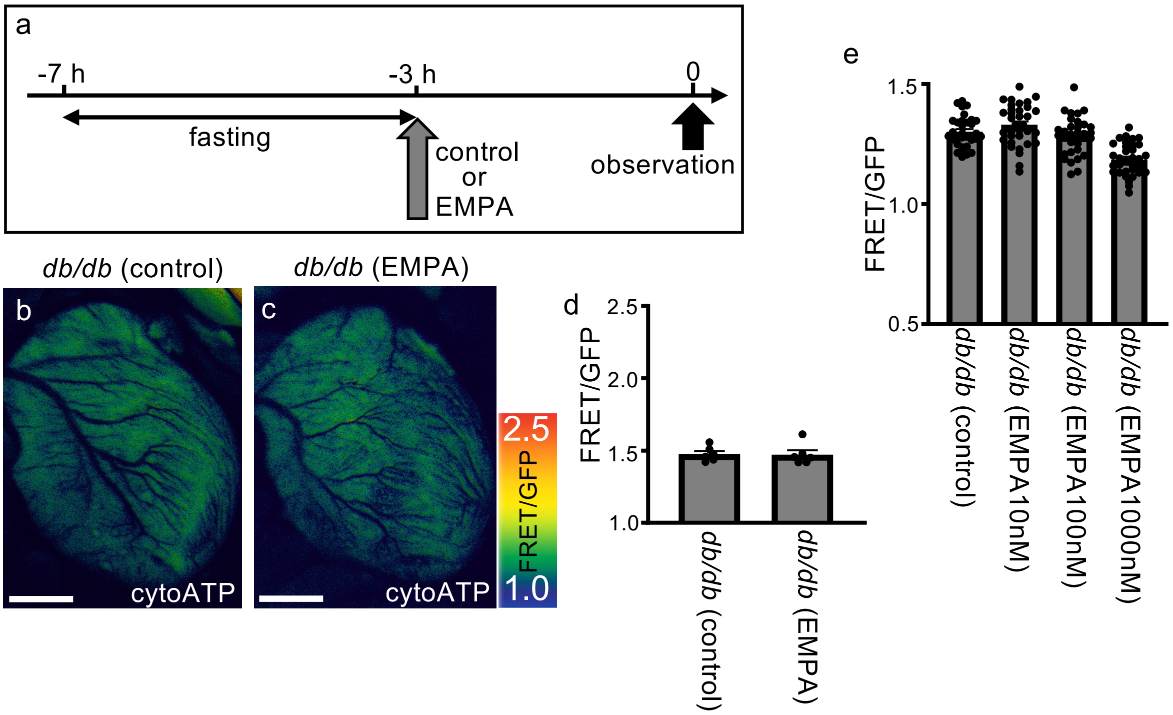

Supplementary Figure 4. Single treatment with empagliflozin did not increase cytosolic ATP levels in the heart of T2D model mice. a Schematic illustration of the treatment regimen (EMPA: empagliflozin). **b-c** Representative fluorescence images of a live heart from each condition. Warmer colors indicate higher ATP concentrations. Scale bar: 2 mm. **d** FRET/GFP ratio indicating the amount of ATP in the heart under each condition (*db*/*db* [control]: n = 6, *db*/*db* [EMPA]: n = 6). There was no significant difference between the control and EMPA groups. **e** The FRET/GFP ratio was examined 1 h after the addition of control (1.3 ± 0.006, n = 32) or 10 nM (1.3 ± 0.009, n = 34), 100 nM (1.3 ± 0.007, n = 33) or 1000 nM (1.2 ± 0.011, n = 34) empagliflozin (EMPA) to mature cardiomyocytes isolated from 8-week-old *db/db*; cytoATP-Tg mice. There was no significant difference between the control and EMPA groups.
